# Evaluation of different safety-engineered protection mechanisms of port access needles using a lifelike model of vascular access routes

**DOI:** 10.3389/fmedt.2025.1505184

**Published:** 2025-04-03

**Authors:** Feline Gabler, Pierre Heiden, Peter Deibert, Daniel Steinmann

**Affiliations:** ^1^ Department of Anesthesiology and Critical Care, Medical Center—University of Freiburg, Freiburg, Germany; ^2^ Occupational Medical Service, Medical Center—University of Freiburg, Freiburg, Germany; ^3^ Institute of Exercise and Occupational Medicine, Medical Center—University of Freiburg, Freiburg, Germany

**Keywords:** port access needles, needlestick injury, safety-engineered protection mechanism, lifelike model, medical students

## Abstract

**Background:**

Preventing needlestick injuries caused by hypodermic needles is crucial for healthcare personnel. In this context, port access needles play an important role. However, systematic comparisons of different safety-engineered port access needles have not been conducted. Therefore, we evaluated differences in product characteristics and user preferences of safety-engineered protection mechanisms of port access needles.

**Methods:**

Port puncture was performed using port access needles with four different safety mechanisms: (a) EZ Huber™ PFM Medical, (b) Gripstick® Safety OMT, (c) Gripper Micro® Smiths Medical and (d) pps ct® Vygon. Each needle type was used in three consecutive tries: an uninstructed first handling, after which instructions were given according to operating manual. Subsequently, a first and second trial were conducted. Study endpoints included successful activation, activation time, way of activation (one hand or two hands), correct activation, possible risk of needlestick injury, possibility of deactivation and preferred safety mechanism.

**Results:**

Overall, successful activation rate during the second trial was equal for all four devices (100%). Median activation time was (a) 6 s, (b) 3 s, (c) 11 s and (d) 6 s. Single-handed activation during the second trial was (a) 0%, (b) 75%, (c) 1% and (d) 1%. Single-handed activation after further preparation with two hands during the second trial was (a) 0%, (b) 0%, (c) 0% and (d) 50%. Correct activation during the second trial was (a) 97%, (b) 66%, (c) 19% and (d) 44%. Possible risk of needlestick injury during the second trial was highest with (b). Possibility of deactivation was (a) 75%, (b) 94%, (c) 97% and (d) 22%. Individual preferences for each system were (a) *n* = 5, (b) *n* = 2, (c) *n* = 1 and (d) *n* = 24. The main written reasons given for preference were the safety protection mechanism and handling of the port needle.

**Conclusion:**

We have shown significant differences regarding product characteristics of safety mechanisms of port access needles. Our evaluation approach provides specific data for both, technical (e.g., single-handed activation) and personal device selection criteria (e.g., preference of the safety mechanism).

## Introduction

Port access needles, used for accessing implanted ports, play a crucial role in providing reliable and convenient access to the vascular system (1). Despite their significance, issues related to safety and the potential for needlestick injuries remain a concern (2–4). In this context, various safety-engineered protection mechanisms have been introduced in the clinical setting to solve the general problem of needlestick injuries (5). Nevertheless, needlestick injuries still occur, even after education and training with devices containing safety-engineered protection mechanisms (6). Looking at factors affecting the occurrence of needlestick injuries on the level of tool and technology factors, the use of personal protective equipment had the highest relative weight followed by the safety design of devices (7).

For designing safety-engineered protection mechanisms, detailed specifications such as the ability to activate the device with one hand are described by current regulations (8–10). To date, a wide range of devices with safety-engineered protection mechanisms have been introduced, including blood collection needles, winged blood collection needles, peripheral intravenous catheters and port access needles. Several studies have been conducted to evaluate different types of safety-engineered protection mechanisms (2, 11–14). These investigations repeatedly found that most injuries occur before or even during activation of the safety-engineered protection mechanism, highlighting the impact of the mechanism itself on the prevention of needle-stick injuries and the need for ongoing optimization of safety-engineered protection mechanisms (15–19).

In the context of available frameworks for implementation of sharp injury preventing programs (8, 10, 20), we proposed a systematic model-based user evaluation of devices with safety-engineered protection mechanism prior to clinical implementation (21). To date, only few user-acceptability studies prior to introduction of safety-engineered port access needles into the clinical area have been published (22–24). New promising approaches focus on virtual reality and corresponding haptic simulation methods, enabling training, evaluation and design optimizations (25–28); however, virtual reality technology is still challenging to simulate fine motor interactions (29). In a previous study, the Polyperf® Safe (PPS) Huber needle was evaluated in cancer patients (22). Compared to the standard Gripper® needle in this study, most nurses were convinced that the PPS needle was safer than the Gripper® needle. However, this study was solely based on questionnaire evaluations with no further information regarding safety aspects. Hence, a systematic comparison of different safety-engineered port access needles and their underlying fundamental mechanisms has not been conducted. Therefore, we expanded our model-based user evaluation of devices with safety-engineered protection mechanism using a lifelike simulation model for port access needles.

In this randomized lifelike model-based study, we hypothesized that significant differences in product characteristics and inexperienced healthcare personnel would reveal user preferences of safety-engineered protection mechanisms of port access needles.

## Materials and methods

### Participants and ethics

The study was approved by the local Medical Research Ethics Committee Freiburg (Research Ethics Committee Reference Number: 44/14). Third-year medical students from the University Medical School Freiburg (Germany) were selected randomly using a standard random generator (Microsoft Excel) and invited to participate in the study. Exclusion criteria included prior routine experience with port puncture and safety-engineered port needles (e.g., prior employment as a nurse or physician assistant). The participants had to give their informed written consent to be tested and analysed.

### Port needles

**Figure 1:**
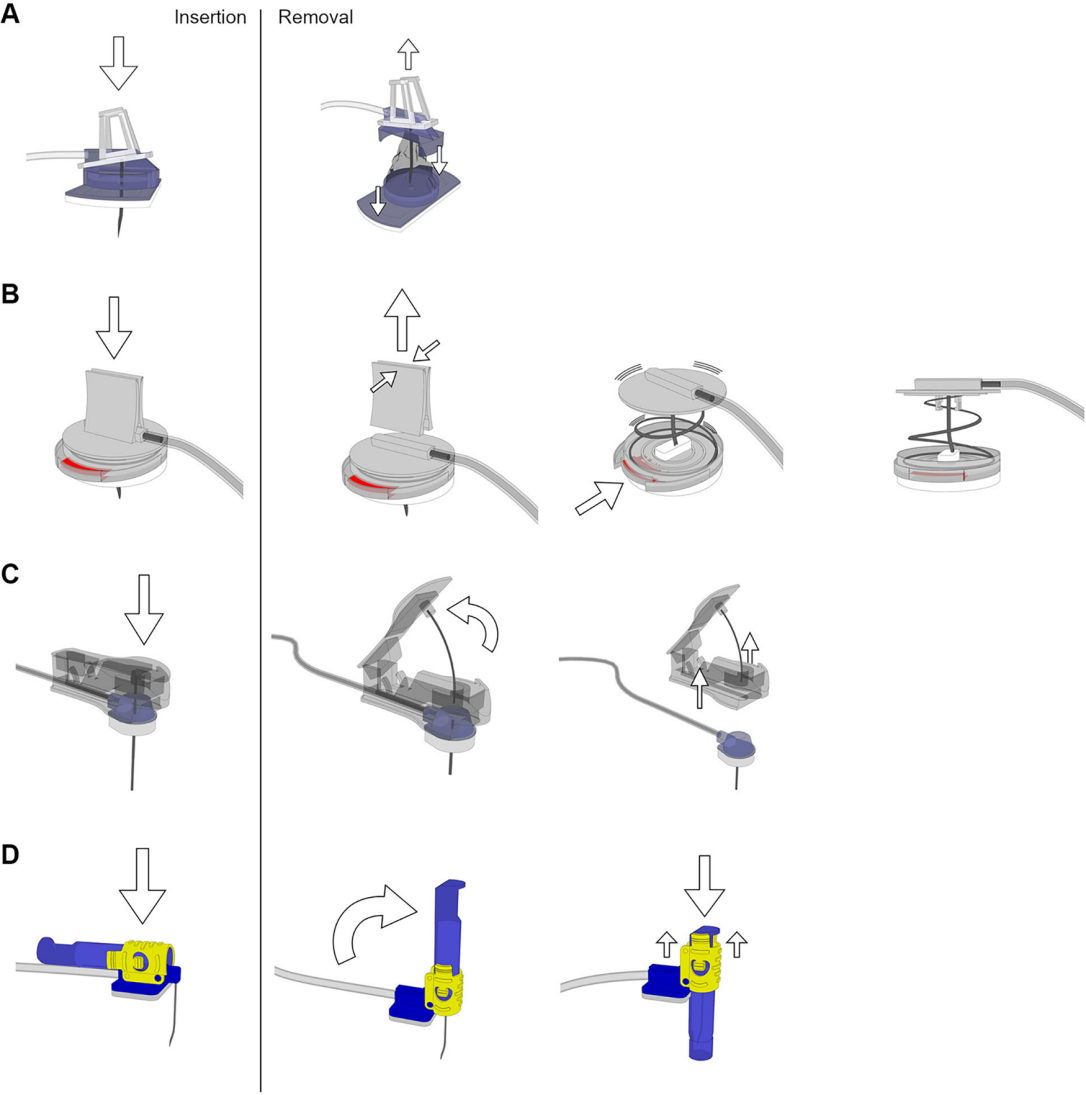
Port needles with different safety-engineered protection mechanisms. Handling while insertion and removal of the needles: **(A)** EZ Huber™ PFM Medical (needle pulled into a protective cover on removal), **(B)** Gripstick® Safety OMT (spring retraction of the needle via the release button), **(C)** Gripper Micro® Smiths Medical (removal of the needle in conjunction with the safety component after insertion) and **(D)** pps ct® Vygon (protective cover pushed over the needle during removal). Images have been created by 3D modeling and rendering using Autodesk Maya 2012 (Autodesk Inc., San Rafael, USA) and Adobe Photoshop (Adobe Inc., San José, USA).

Four different port needles representing four different safety-engineered protection mechanisms were tested (Figure 1): i.e., (a) EZ Huber™ PFM Medical (pfm medical gmbh, Köln, Germany) = protector slipped over the needle while pulling out, (b) Gripstick® Safety OMT (OMT GmbH & Co. KG, Frittlingen, Germany) = needle protector closed over the needle via spring mechanism after pressing the release button, (c) Gripper Micro® Smiths Medical (Smiths Medical Deutschland GmbH, Grasbrunn, Germany) = two parted mechanism which removes the safety part and leaves a blunt cannula: while removing the safety part, a protection snaps into place, and (d) pps ct® Vygon (Vygon, Aachen, Germany) = protective cover pushed over the needle while withdrawing.

### Study protocol

**Figure 2:**
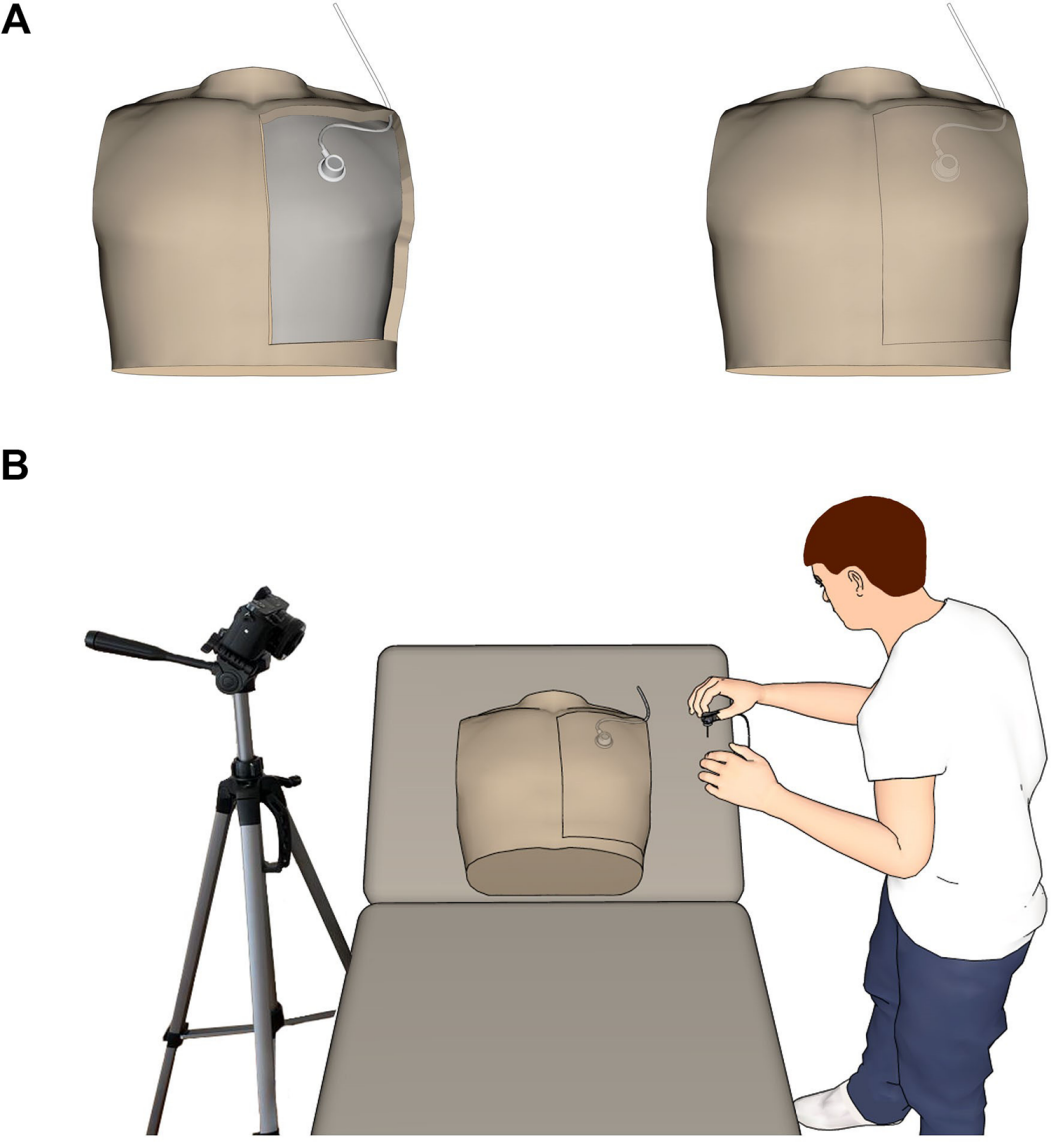
Experimental set-up for port needle evaluation using the Chester Chest™ lifelike model of common long-term vascular access routes. **(A)** Placement of the intravascular access device within the torso. **(B)** Positioning of the model and the video camera during the puncture trial. Images have been created by 3D modeling and rendering using Autodesk Maya 2012 (Autodesk Inc., San Rafael, USA) and Adobe Photoshop (Adobe Inc., San José, USA).

The experimental set-up for port puncture included a simulation model (“Chester Chest”, Laerdal Medical GmbH, Puchheim, Germany) and a commercial video documentation camera (Sony Alpha 6400, Sony Europe B.V., Berlin) for *post-hoc* analysis of each puncture attempt (Figure 2). At first, all participants were instructed to perform each port puncture as follows: wearing gloves, skin disinfection, preparation of the port needle, port puncture, position control, decannulation and subsequent activation of the specific safety-engineered protection mechanism. Three consecutive attempts were recorded: an uninstructed first handling, followed by instruction according to the manufacturer's operating manual, followed by a first and a second trial. The order in which the four different port needles were used was randomized for all participants. For the uninstructed first handling, participants were asked to perform a port puncture immediately after randomization. This shows whether the safety-engineered protection mechanism is self-explanatory or not. After the second trial, participants were asked to deactivate the safety-engineered protection mechanism. Subsequently, the participants had to answer a questionnaire on their experience with each safety-engineered protection mechanism of the four port needles using a Likert score (1 = strongly agree; 2 = agree; 3 = neutral; 4 = disagree; 5 = strongly disagree).

### Study endpoints

Endpoints analyzed by video included successful activation of the safety-engineered protection mechanism, the time required for activation, ability to execute one- or two-handed activation, correct activation of the safety mechanism, the risk of needlestick injury, troubles with handing before starting the puncture, the possibility of deactivation and the preferred safety-engineered protection mechanism. The time required for activation was defined as the time required from decannulation of the needle tip outside the skin (EZ Huber™ PFM Medical, Gripper Micro® Smiths Medical, and pps ct® Vygon) or contact of the fingers with the release slide (Gripstick® Safety OMT) until complete activation of the safety-engineered protection mechanism. Correct activation was defined according to the manufacturer's instruction. Risk of possible needlestick injury was defined as one finger coming within a distance of less than 1 cm next to the tip of the needle prior to activation of the safety-engineered protection mechanism. Troubles with handling before starting the puncture were defined for taking more than 2 s or even failing to remove the needle protection cap. Possible deactivation was defined as a free needle tip after maximum manipulation of the activated safety-engineered protection mechanism.

### Statistical analysis

Nonparametric data were tested for differences using Cochrane's Q test followed by McNemars's exact test and Friedman's test followed by Wilcoxon's signed rank sum test if indicated. Data from “not possible activation of safety-engineered protection mechanism trials” were excluded from calculation of activation times. GraphPad Prism® 9.2.0 for Microsoft Windows (GraphPad Software Inc., La Jolla, California, USA) and MedCalc® 20.014 for Microsoft Windows (MedCalc Software bvba, Ostend, Belgium) were used for statistical analysis. A *P*-value of <0.05 was chosen as the level of significance. In cases of multiple comparisons, *P* was corrected using Bonferroni's approach for posthoc tests resulting in a *P*-value of <0.0083 being considered significant.

## Results

From 300 third-year medical students, 32 were randomly selected and all of them consented to participate in this study. Of the enclosed participants, 22 were women. The average age was 26 years.

### Video analysis

Results from the port puncture simulations were summarized in Table 1. Overall successful activation rate for uninstructed first handling was best for EZ Huber™ PFM Medical (84%), followed by pps ct® Vygon (56%), Gripper Micro® Smiths Medical (41%) and Gripstick® Safety OMT (31%). Overall, successful activation rate improved during the second trial for all the devices (100%). Median time required for safety mechanism activation was shorter for Gripstick® Safety OMT compared to the other three devices (*P* < 0.0083). Compared to the other devices, activation with one hand during the second trial was significantly higher with Gripstick® Safety OMT (75%) (*P* < 0.0083) and higher for pps ct® Vygon (50%) for activation with one hand after further preparation with two hands (*P* < 0.0083). Correct activation of the safety mechanism during second trial was higher for EZ Huber™ PFM Medical (97%) compared to Gripper Micro® Smiths Medical (19%) and pps ct® Vygon (44%) and Gripstick® Safety OMT (66%) compared to Gripper Micro® Smiths Medical (19%) (*P* < 0.0083) and increased for all four devices after instruction compared to first handling (*P* < 0.05). All four devices could protect from risk of possible needlestick injury during the second trial. Trouble with handling before starting the puncture for uninstructed first handling (91%) and the first trial (19%) occurred while using the Gripstick® Safety OMT due to difficulties in removing the needle protection cap (*P* < 0.0083). Deactivation of safety mechanism was possible with Gripper Micro® Smiths Medical (97%), Gripstick® Safety OMT (94%), EZ Huber™ PFM Medical (75%) and pps ct® Vygon (22%). Premature activation occurred only with Gripstick® Safety OMT during first handling (34%) and first trial (3%).

**Table 1:** Summary of the results from the port puncture simulation.

Parameter assessed	EZ Huber™ PFM Medical	Gripstick® Safety OMT	Gripper Micro® Smiths Medical	pps ct® Vygon
Successful activation of the safety mechanism; *n* (%)				
1st handling	27 (84)**^,^***	10 (31)*	13 (41) *	18 (56)
1st trial	32 (100)	31 (97)	31 (97)	32 (100)
2nd trial	32 (100)	32 (100)	32 (100)	32 (100)
Median successful activation time (1st + 2nd trial), sec (IQR)	6 (4–10)**^,^***	3 (1–5)*^,^***^,^****	11 (6–16)*^,^**^,^****	6 (4–10)**^,^***
Way of activation: one hand, two hands or one hand after further preparation with two hands; *n* (%)				
1st handling	0/27 (84)/0**^,^***	3 (9)/7 (22)/0*^,^****	0/13 (41)/0*	0/18 (56)/0**
1st trial	0/32 (100)/0**^,^****	20 (63)/11 (34)/0*^,^***^,^****	0/32 (100)/0**^,^****	0/17 (53)/15 (47)*^,^**^,^***
2nd trial	0/32 (100)/0**^,^****	24 (75)/8 (25)/0*^,^***^,^****	1 (3)/31 (97)/0**^,^****	1 (3)/15 (47)/16 (50)*^,^**^,^***
Correct activation of the safety mechanism; *n* (%)				
1st handling	10 (31)****	2 (6)	1 (3)	0 *
1st trial	30 (94)**^,^***^,^****	18 (56)*^,^***	5 (16)*^,^**	12 (38) *
2nd trial	31 (97)**^,^***^,^****	21 (66)*^,^***	6 (19)*^,^**	14 (44) *
Risk of possible needlestick injury *n* (%)				
1st handling	15 (47)****	11 (34)***^,^****	24 (75)**	28 (88)*^,^**
1st trial	0	2 (6)	2 (6)	0
2nd trial	0	1 (3)	0	0
Trouble with handling before starting the puncture; *n* (%)				
1st handling	0**	29 (91)*^,^***^,^****	0**	0**
…1st trial	0**	6 (19)*^,^***^,^****	0**	0**
…2nd trial	0	1 (3)	0	0
Deactivation of the safety mechanism possible (2nd trial); *n* (%)	24 (75)****	30 (94)****	31 (97)****	7 (22)*^,^**^,^***
Premature activation (1st handling + 1st trial + 2nd trial); *n* (%)	0**	12 (12,5)*^,^***^,^****	0**	0**

* *P* < 0.0083 vs. EZ Huber™ PFM Medical, **P < 0.0083 vs. Gripstick® Safety OMT, ***P < 0.0083 vs. Gripper Micro® Smiths Medical, ****P < 0.0083 vs. pps ct® Vygon.

### Individual written evaluation

Results from the questionnaire are summarized in Table 2. Compared to the other devices the pps ct® Vygon was rated best for ease of determining activation, effectiveness in reducing needlestick injuries, impossibility of deactivation and operator safety (*P* < 0.0083). Gripstick® Safety OMT and pps ct® Vygon were rated best for activation using one hand (*P* < 0.0083). Gripper Micro® Smiths Medical was rated worst for no training needed for use and ease of handling after being briefed on the user's manual (*P* < 0.0083). No differences were found between the devices regarding the visualization of the needle tip. Twenty-four medical students preferred the pps ct® Vygon needle, five the EZ Huber™ PFM Medical, two the Gripstick® Safety OMT and one the Gripper Micro® Smiths Medical (Table 2).

**Table 2:** Summary of the results from the questionary. Results are taken as Likert score (1 = strongly agree; 2 = agree; 3 = neutral; 4 = disagree; 5 = strongly disagree). Values are reported as median (IQR).

Parameter Assessed	EZ Huber™ PFM Medical	Gripstick® Safety OMT	Gripper Micro® Smiths Medical	pps ct® Vygon
The safety-engineered protection mechanism …				
is easy to activate	1 (1–2,75)***	2 (1–3)	3 (2–3,75)*^,^****	1 (1–2)***
is intuitive to use	2 (1–3)***	3 (2–4)	4 (3–5)*	3 (2–4)
could be activated using one hand	5 (4–5)**^,^****	1 (1–1)*^,^***	4 (4–5)**^,^****	2 (1–4)*^,^***
did not hinder routine use	1 (1–2)	2 (1–2,75)****	1 (1–2)	1 (1–2)**
does not restrict visualization of the tip of the needle	1 (1–2)	2 (1–3)	1 (1–3)	1 (1–2)
is easy to determine when it has been activated	3 (2–4)**^,^****	1 (1–2)*^,^****	2 (1,25–3)****	1 (1–1)*^,^**^,^***
doesn`t need training to be used	2 (2–4)***	4 (2–4)***	4 (3,25–5)*^,^**^,^****	3,5 (2–4)***
would be effective in reducing needlestick injury	2 (1–3,75)****	3 (2–4)****	2 (1,25–3)****	1 (1–2)*^,^**^,^***
could not be easily deactivated	4 (2–5)****	5 (4–5)****	4 (3–5)****	1 (1–2)*^,^**^,^***
is easy to handle after work through the user manual	2 (1–2)**^,^***	1 (1–3)*^,^***	3 (2–4)*^,^**^,^****	2 (1–2)***
is safe for operators	2 (1–3)****	2,5 (2–4)****	3 (2–3)****	1 (1–2)*^,^**^,^***
Preference of the safety mechanism *n* (%)	5 (16)****	2 (6)****	1 (3)****	24 (75)*^,^**^,^***

* *P* < 0.0083 vs. EZ Huber™ PFM Medical, **P < 0.0083 vs. Gripstick® Safety OMT, ***P < 0.0083 vs. Gripper Micro® Smiths Medical, ****P < 0.0083 vs. pps ct® Vygon.

Overall text comments for preference of one of the four port needles included: (i) safest protection mechanism (*n* = 13), (ii) the port access needle is best to handle (*n* = 13), and (iii) the mechanism cannot be deactivated (*n* = 11).

## Discussion

The main ﬁndings of this study can be summarized as follows: (i) the overall successful activation rate during the second trial was equal for all devices, (ii) the median time required for safety mechanism activation was shortest for Gripstick® Safety OMT, (iii) single-handed activation during the second trial was best for the Gripstick® Safety OMT, (iv) the risk of possible needlestick injury during second trial was equal for all devices, (v) trouble with handling before starting the puncture including premature activation was highest for Gripstick® Safety OMT, (vi) deactivation of the safety mechanism was lowest for pps ct® Vygon, and (vii) users preferred the most comprehensive safety-engineered protection mechanism and a mechanism that cannot be deactivated.

As shown for other devices (21), the overall successful activation rate was high and equal for all port access needles. However, the median time required for safety mechanism activation was shortest for Gripstick® Safety OMT with 3 s vs. 6–11 s for the other needles. Single-handed activation during the second trial was also best for Gripstick® Safety OMT with 75% vs. 0%–3% for the other needles. These characteristics may provide an additional level of safety in day-to-day clinical settings. The notable proficiency in activating the safety mechanism, particularly with EZ Huber™ PFM Medical and Gripstick® Safety OMT, implies that the other two safety mechanisms may need more comprehensive instructions for correct use. We have identified a potential issue with the Gripstick® Safety OMT concerning inadvertent premature activation when used without proper guidance. Consequently, none of the assessed safety mechanisms was fully self-explanatory.

Several studies repeatedly found that most injuries occur before or even during activation of the safety-engineered protection mechanism, highlighting the impact of the mechanism itself on the prevention of needle-stick injuries and the need for ongoing optimization of safety-engineered protection mechanisms (15–19). Our findings show that all four devices could protect from risk of possible needlestick injury during the second trial with almost no identifiable risk of possible needlestick injury. One safety-engineered protection mechanism (Gripstick® Safety OMT) showed significant trouble with the handling before starting the puncture during first handling and first trial due to premature activation of the spring mechanism by unintentionally pressing the release button during removal of the needle protection cap. Therefore, our findings could lead to an improvement of the current mechanisms as well as for the future designs of safety-engineered protection mechanisms.

All healthcare personnel, including students and trainees, should be educated and trained in locally available safety-engineered protection devices with a priority on educational interventions in high-risk settings (6, 10). However, if education and training have not been carried out, safety-engineered protection mechanisms should be as self-explanatory as possible (21). We have identified several problems during uninstructed first handling: the activation with two hands in most cases, a possible risk of needlestick injury with almost all devices, several premature activations and troubles with handling before the puncture with one device. This information may aid in optimizing designs and identifying safety-engineered protection devices that are self-explanatory.

As concluded in an analysis of safety-engineered protection mechanisms of winged blood collection needles (21), Jagger and Perry highlighted the crucial involvement of healthcare workers in selecting safety-engineered devices (9). Adams and Elliott suggested evaluating safety-engineered needle devices before introduction, recognizing that no single device can satisfy all requirements or preferences of healthcare workers (13). Our findings indicate that healthcare personnel are not only capable of assessing various devices with safety-engineered protection mechanisms, as demonstrated previously (21), but also capable of offering detailed insights into their preferred devices and the reasons behind their choices. Moreover, our study suggests a preference for needle retraction devices (such as pps ct® Vygon) over needle shielding devices (such as EZ Huber™ PFM Medical and Gripstick® Safety OMT), as previously shown for winged blood collection needles (21). These results are in line with a prior study where most nurses believed the PPS needle was safer than the traditional Deltec™ Gripper® needle (22).

In practice, there are sometimes disagreements about which criteria are decisive for selecting a certain device. To address this issue, relevant findings can further be presented using comprehensive visualization methods (see Supplemental Materials: Graphs S1 and S2). Furthermore, our approach could be integrated into a comprehensive procurement framework within various healthcare facilities. We recommend a prioritization of the following fundamental criteria: successful activation rate, risk of potential needlestick injury and preference. This may ensure a high user compliance rate and add additional safety during the disposal process of hypodermic needles.

In addition, the specific design characteristics and differences between the four evaluated products may explain some of the observed differences in handling and efficacy. The EZ Huber™ PFM Medical is a modified version of a traditional needle, incorporating the safety-engineered protection mechanism into an established product. The Gripstick® Safety OMT represents a push button approach with a focus on ease of activation. In contrast, the Gripper Micro® Smiths Medical was designed to minimize the size of the port needle itself while in place on the patient, in combination with a safety-engineered protection mechanism. The pps ct® Vygon can be interpreted as a partially compromise between size and a sophisticated safety-engineered protection mechanism. In this context, virtual reality and corresponding haptic simulation methods (25–28) may enable the study of other testing parameters, such as patient movement or other difficulties during insertion/removal, as well as simulation of new developed safety-engineered protection mechanism principles during prototyping and prior to application in patients.

One limitation of the study is the lifelike model of vascular access routes, as in other simulation studies (21), complicating factors like different nature of skin quality, bleeding, patient behaviour such as movement of the chest during port cannulation or stress of the unexperienced user during the procedure cannot be simulated reliably. We did not investigate potential additional training effects beyond three attempts with each port access needle, nor did we assess the impact of an accompanying education and training program on preferences, as suggested (6, 10). Our study also did not analyse the effect on needlestick injury rates; thus, it does not establish the actual safety performance of the various devices. Instead, our focus was on the features of the safety-engineered protection mechanism itself and its usability, particularly when utilized by inexperienced and minimally trained healthcare personnel.

## Conclusion

In summary, our research has revealed substantial variations among inexperienced healthcare workers in their perception of safety-engineered port access needle features. Specifically, we found that the preference for the most comprehensive safety mechanism was a key factor, with devices incorporating needle retraction being favoured over those employing needle shielding. Furthermore, our evaluation approach provides specific data for technical device selection criteria (e.g., single-handed activation), which are crucial for hypodermic needles. Consequently, we propose our lifelike model-based study as a potential tool for evaluating new healthcare devices before clinical deployment, aiding in the integration of safer needle designs into healthcare settings.

## Data Availability

The raw data supporting the conclusions of this article will be made available by the authors, without undue reservation.

## Supplementary material

Graph 1 Visualization of the results from the port puncture simulation for comparison of the four port needles with different safety-engineered protection mechanisms using enhanced circular layout generation (RS1).

Graph 2 Visualization of the results from the questionnaire for comparison of the four port needles with different safety-engineered protection mechanisms using enhanced circular layout generation (RS1).
