# Supplementary figures and images for: Evaluation of different safety-engineered protection mechanisms of port access needles using a lifelike model of vascular access routes
Source: Front Med Technol. 2025 Apr 3;7:1505184. doi: 10.3389/fmedt.2025.1505184 (PMC12003311; doi:10.3389/fmedt.2025.1505184)

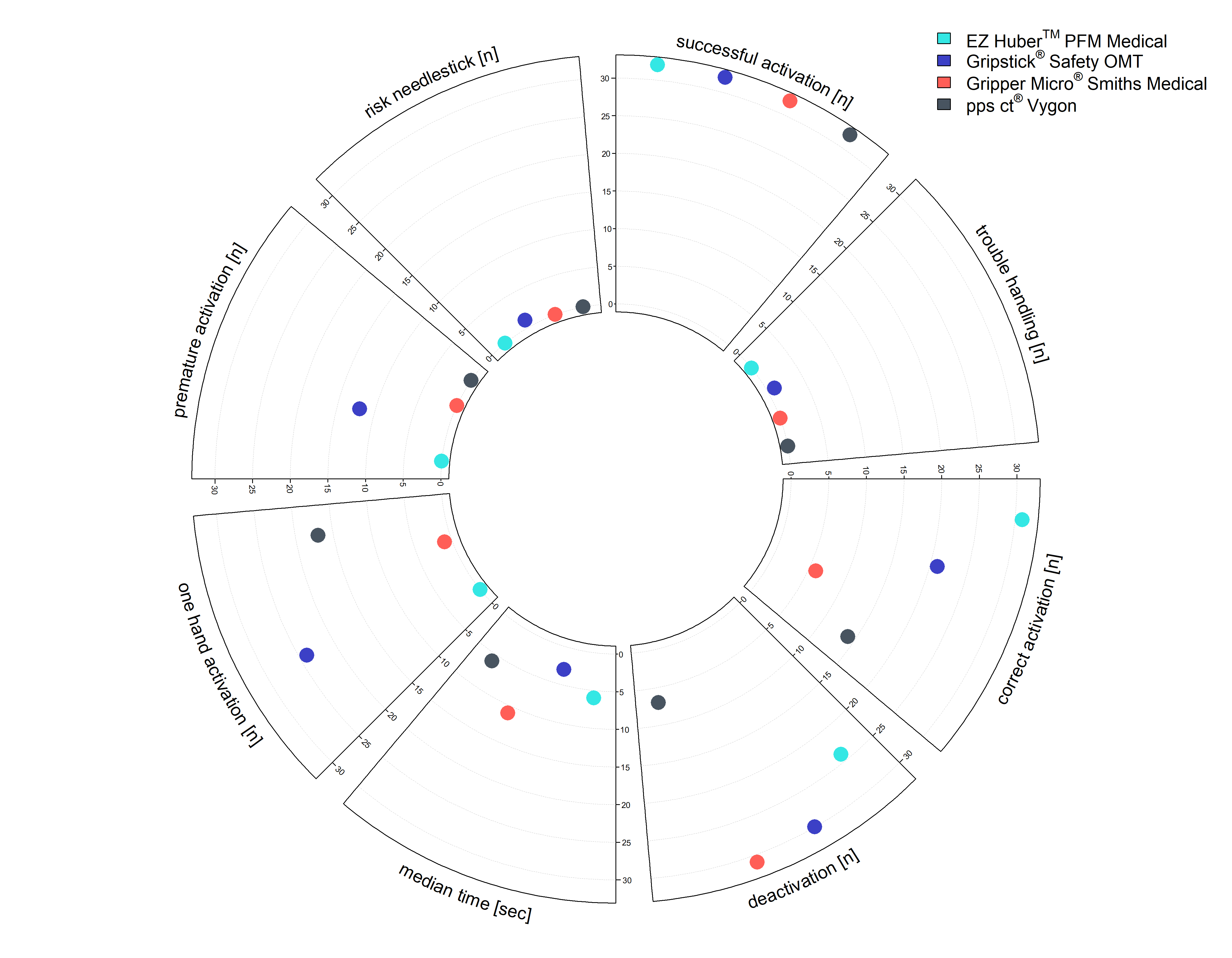

Supplement: Graph 1 — Visualization of the results from the port puncture simulation for comparison of the four port needles with different safety-engineered protection mechanisms using enhanced circular layout generation (RS1). [file Image1.png]

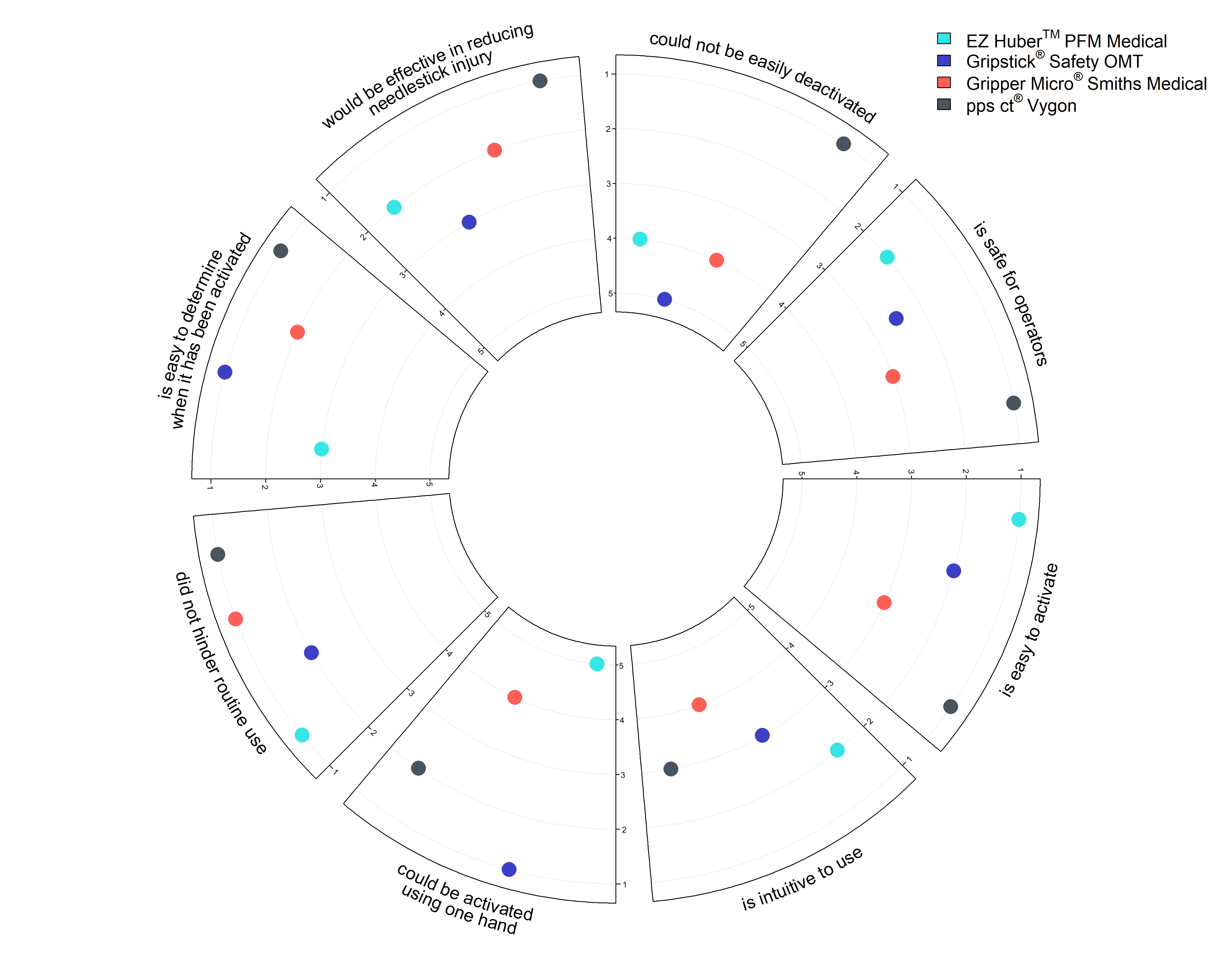

Supplement: Graph 2 — Visualization of the results from the questionnaire for comparison of the four port needles with different safety-engineered protection mechanisms using enhanced circular layout generation (RS1). [file Image2.png]
