# Supplementary material for: Evaluation of different safety-engineered protection mechanisms of port access needles using a lifelike model of vascular access routes
Source: Front Med Technol. 2025 Apr 3;7:1505184. doi: 10.3389/fmedt.2025.1505184 (PMC12003311; doi:10.3389/fmedt.2025.1505184)
Supplement: Supplementary file 3 [file Datasheet1.docx]

SUPPLEMENTARY MATERIAL - REFERENCES

RS1. Gu Z, Gu L, Eils R, Schlesner M, Brors B. circlize implements and enhances circular visualization in R. Bioinformatics. (2014) 330(14):2811-2812. doi: 10.1093/bioinformatics/btu393
